# Digital ankle-brachial index technology used in primary care settings to detect flow obstruction: a population based registry study

**DOI:** 10.1186/1756-0500-6-404

**Published:** 2013-10-08

**Authors:** Tiffini R Diage, Gayle Johnson, Gowtam Ravipati

**Affiliations:** 1 4050 Olson Memorial Hwy, Suite 450, Minneapolis, MN 55422, USA; 2 6957 W Plano Pkwy, Suite 1000, Plano, TX 75093, USA

**Keywords:** Ankle-brachial index, Peripheral artery disease, Flow obstruction

## Abstract

**Background:**

Peripheral artery disease affects 8–18 million people in the United States. Patients with peripheral artery disease are known to have increased morbidity and mortality. Medical guidelines recognize ankle-brachial index testing as an effective screening tool that allows for early detection of this disease in primary care settings. Doppler ankle-brachial index, the standard method used, is time consuming and requires technical expertise. Automated (digital) ankle-brachial index testing through plethysmography may be a more attractive method in primary care settings due to its speed and ease of use. This observational study evaluated the use of one digital ankle-brachial index device in primary care settings to describe the population tested and the results obtained.

**Results:**

A total of 19 medical practices throughout the United States provided data on 632 patient tests. In the population tested, the mean age was 67.2 (±13.8) years, and 38% of patients were male. Additionally, 94.7% of the population had risk factors, signs and/or symptoms suspicious for peripheral artery disease, and 20.3% presented with claudication. Twelve percent (76/632) of patient tests showed an abnormal digital ankle-brachial index (<0.93), indicating a result positive for peripheral artery disease; the frequency of hypercholesterolemia, hypertension, and coronary artery disease in this group was 62% (45/73), 69% (50/72) and 46% (34/74), respectively.

**Conclusion:**

The results of this study support the use of a digital ankle-brachial index device using blood volume plethysmography technology for evaluation of peripheral artery disease. Data is consistent with previously reported population characteristics with respect to peripheral artery disease prevalence, signs/symptoms, and risk factors. The device used in this study enabled evaluation for peripheral artery disease in primary care settings and may allow for early detection of the disease.

## Background

Peripheral artery disease (PAD) affects between 8–18 million people in the United States [1].Patients with PAD are known to have increased morbidity and mortality [2,3]. Progression of PAD directly results in claudication, impaired walking, and amputation. The incidence of stroke, myocardial infarction, death or hospitalization is over 20% for patients with PAD [4]. Numerous medical guidelines recognize early detection of PAD as a critical factor both for mitigating PAD progression and improving risk management of cardiovascular disease [5-7].

PAD is characterized by impaired blood flow in the peripheral arteries due to atherosclerosis. Reduced blood flow and endothelial dysfunction are significant contributors to the progression of this disease, and the body of research on genetic factors involved is continually growing [8,9]. Impaired angiogenesis and decreased capillary supply to skeletal muscle are seen in PAD and may be related to the symptoms and reduced functionality experienced by these patients. A recent trial showed that after participating in a supervised exercise training regimen, PAD patients experience an increase in microvasculature in the calf skeletal muscle (as measured by capillary density) and subsequent improvement in exercise capacity [10]. While possibly not the only mechanism for improved functionality, this study suggests the microvasculature involved may have a significant impact on the metabolic potential of working muscles in PAD patients. Research on potential treatments based on these mechanisms of action is ongoing, and early detection of PAD remains very important in the treatment course of patients with this disease.

Ankle-brachial index (ABI) testing is recommended by the US Preventative Services Task Force and American College of Cardiology for evaluating patients at risk for PAD [6,7]. ABI testing in primary care settings can provide early diagnosis of PAD, allowing for earlier treatment [11-13]. There are several methods used to perform ABI measurement, with Doppler ABI being most common. However, adoption of Doppler-based ABI in primary care is limited. Previous studies have reported barriers to implementation, including the time required to conduct tests, the training needed, and the technical skills required [12,14].

Automated ABI, either through oscilloscope or plethysmography, may be a more attractive option in primary care settings. This method is faster and easier, while maintaining accuracy [11,12]; clinical results comparing alternative ABI methods to Doppler ABI have shown concordance above 90% [15-18]. This study evaluated the use of a specific plethysmography, or digital ABI, device in primary care settings to determine if results were consistent with known PAD population characteristics.

## Methods

A device-specific, voluntary data registry was created to capture physician reported results on adult patients. A standardized, self-administered questionnaire was used to identify PAD signs, symptoms and cardiovascular risk factors in patients presenting to primary care practices (see Additional file 1). Patients with signs, symptoms and/or risk factors for PAD were then tested with a digital ABI device (FloChec™ manufactured by Semler Scientific, Inc, Portland Oregon United States) to evaluate flow obstruction in each lower extremity compared to the corresponding upper extremity. All data was de-identified. ABI results, PAD signs and symptoms, and cardiovascular risk factors were recorded and input into a central database for analysis.

The FloChec™ device is a blood volume plethysmography technology that received Food and Drug Administration (FDA) clearance for commercial use in the United States in 2010. The FloChec™ system consists of a sensor coupled with an infrared light emitting diode (LED) within a pulse-oximetry style clamp, and an attached portable computer that runs proprietary software. Measurements with FloChec™ are performed bilaterally on the lower and upper extremities by placing the clamp on one digit of each extremity in sequential fashion. During measurement, the LED transmits light into the digit, and the sensor measures reflected/scattered light. The blood volume in the digit affects the amount of returning light, and these blood volume waveforms are recorded and analyzed by the device. Blood flow index (BFI), a measure of proximal patency, is calculated by a proprietary algorithm for each extremity. Comparison of BFI in the lower extremity to BFI in the upper extremity produces the ratio known as digital ABI. Four pulse waveforms, four blood flow indices, and two ABI results are provided in a standard report for each patient. A digital ABI result of <0.93 indicates the presence of flow obstruction.

Binary variables were created for each sign, symptom, or risk factor based on the corresponding questionnaire response. A subject was considered to have claudication if the response to question one was “no” and the response to questions two and three was “yes”. Standard summary statistics were calculated for study variables of interest. For continuous variables, statistics included the number of observations (N), mean, standard deviation, and 95% confidence interval. Categorical variables were summarized in frequency distributions. Prevalence odds ratios with 95% confidence intervals were calculated for each categorical variable of interest, comparing the PAD positive group to the PAD negative group. A multivariate regression examining the effect of risk factors, signs and symptoms, sex, and age on ABI was conducted. To identify important predictors, a step-wise selection procedure that included all possible predictors collected in the study and all possible two-way interactions was executed. Terms were allowed to enter the model with a significance level ≤0.3 and were removed if they had a significance level ≥0.1. Statistical analyses were conducted in SAS version 9.3 (SAS Institute, Cary, N.C.), and graphics were produced in R version 2.11.1. Because the technology used in this study has been commercially cleared in the US and only de-identified data was collected, ethics committee approval was not required.

## Results

A total of 19 medical practices throughout the United States examined 632 patients with the FloChec™ system. Table 1 provides a summary of practice types that participated. These patients were tested based on results of a self-administered questionnaire that indicated risk factors, signs, and/or symptoms suspicious of PAD. Data was provided on digital ABI results for all patients. Of the 632 patient questionnaires collected, 56 had one or more missing data fields. These patients were included in analysis. The number of completed fields used in each calculation is provided in the tables. Population characteristics are provided in Table 2. The average age of the population tested was 67.2±13.8 years, and 38.0% of the population was male (95% CI 33.7%-42.3%). Of the patients tested, 94.7% had risk factors, signs and/or symptoms suspicious for PAD, and 20.3% (95% CI 17.1%-23.6%) presented with claudication.

**Table 1:** Types of participating clinical sites

**Practice type**	**Number of sites % (n/N)**	**Number of patients tested % (n/N)**
General/Family practice	57.9% (11/19)	52.2% (349/632)
Podiatry practice	31.6% (6/19)	31.3% (198/632)
Other	10.5% (2/19)	13.4% (85/632)

**Table 2:** Summary of study population demographics, signs/symptoms, and risk factors

**Measure**	**All subjects**		**PAD negative subjects**		**PAD positive subjects**	
	**Mean ± SD (N)**	**95% CI**	**Mean ± SD (N)**	**95% CI**	**Mean ± SD (N)**	**95% CI**
	**or**		**or**		**or**	
	**% (n/N)**		**% (n/N)**		**% (n/N)**	
Age (years)	67.2 ± 13.8	66.1, 68.3	67.1 ± 13.9	65.9, 68.2	68.2 ± 12.9	65.2, 71.1
	(627)		(551)		(76)	
Age group (years)						
<50	10.7%	8.3%, 13.1%	10.7%	8.1%, 13.3%	10.5%	3.6%, 17.4%
	(67/627)		(59/551)		(8/76)	
50-59	17.7%	14.7%, 20.7%	18.5%	15.3%, 21.8%	11.8%	4.6%, 19.1%
	(111/627)		(102/551)		(9/76)	
60-69	25.5%	22.1%, 28.9%	25.0%	21.4%, 28.7%	28.9%	18.8%, 39.1%
	(160/627)		(138/551)		(22/76)	
70+	46.1%	42.2%, 50.0%	45.7%	41.6%, 49.9%	48.7%	37.4%, 59.9%
	(289/627)		(252/551)		(37/76)	
Male	38.0%	33.7%, 42.3%	36.6%	32.0%, 41.1%	47.1%	35.2%, 58.9%
	(190/500)		(158/432)		(32/68)	
Claudication	20.3%	17.1%, 23.6%	19.7%	16.3%, 23.1%	25.0%	15.0%, 35.0%
	(122/600)		(104/528)		(18/72)	
Discolored/blue legs	21.6%	18.3%, 24.8%	20.6%	17.2%, 24.0%	28.4%	18.1%, 38.7%
	(133/617)		(112/543)		(21/74)	
Non-healing wound	11.4%	8.9%, 13.9%	11.0%	8.4%, 13.6%	14.5%	6.6%, 22.4%
	(71/622)		(60/546)		(11/76)	
At least 1 sign/symptom	42.1%	38.2%, 46.0%	41.2%	37.1%, 45.3%	48.7%	37.4%, 59.9%
	(264/627)		(227/551)		(37/76)	
High cholesterol	61.5%	57.6%, 65.3%	61.5%	57.3%, 65.6%	61.6%	50.5%, 72.8%
	(375/610)		(330/537)		(45/73)	
Hypertension	72.5%	69.0%, 76.1%	73.0%	69.2%, 76.7%	69.4%	58.8%, 80.1%
	(444/612)		(394/540)		(50/72)	
Diabetes	47.3%	43.4%, 51.3%	46.5%	42.3%, 50.7%	53.3%	42.0%, 64.6%
	(291/615)		(251/540)		(40/75)	
Smoking history	40.1%	36.3%, 44.0%	38.7%	34.6%, 42.8%	50.7%	39.4%, 62.0%
	(250/623)		(212/548)		(38/75)	
Stroke/TIA	13.2%	10.6%, 15.9%	11.5%	8.8%, 14.2%	26.0%	16.0%, 36.1%
	(82/620)		(63/547)		(19/73)	
CAD	33.3%	29.6%, 37.0%	31.6%	27.7%, 35.5%	45.9%	34.6%, 57.3%
	(206/618)		(172/544)		(34/74)	
Number of risk factors						
0	7.8%	5.7%, 9.9%	8.2%	5.9%, 10.5%	5.3%	0.2%, 10.3%
	(49/626)		(45/550)		(4/76)	
1	13.9%	11.2%, 16.6%	13.8%	10.9%, 16.7%	14.5%	6.6%, 22.4%
	(87/626)		(76/550)		(11/76)	
2	23.3%	20.0%, 26.6%	24.5%	20.9%, 28.1%	14.5%	6.6%, 22.4%
	(146/626)		(135/550)		(11/76)	
3+	55.0%	51.1%, 58.8%	53.5%	49.3%, 57.6%	65.8%	55.1%, 76.5%
	(344/626)		(294/550)		(50/76)	

**Figure 1:**
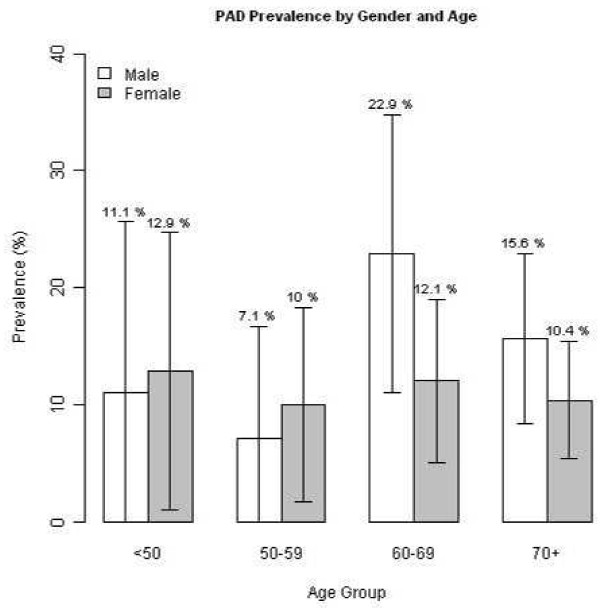
Prevalence of PAD by sex and age group in this study population.

Twelve percent (76/632) of patients had an abnormal FloChec™ result (digital ABI <0.93). The frequency of abnormal left leg result, abnormal right leg result, and abnormal result for both legs was 4.9% (31), 2.8% (18), and 4.3% (27), respectively. Of those with digital ABI <0.93 (PAD positive group), the mean age was 68.2±12.9 years and 47.1% (95% CI 35.2%-58.9%) were male. In this group, hypercholesterolemia, hypertension, and coronary artery disease (CAD) were reported in 61.6% (95% CI 50.5%-72.8%), 69.4% (95% CI 58.8%-80.1%), and 45.9% (95% CI 34.6%-57.3%) of patients, respectively. A self-reported history of smoking was found in 50.7% (95% CI 39.4%-62.0%) of the abnormal digital ABI patients. Figure 1 shows the prevalence of positive PAD result by sex and age group. Patients with digital ABI <0.93 were more likely to be in age groups 60–69 and 70+ (28.9% and 48.7%, respectively) than in age groups <50 and 50–59 (10.5% and 11.8%, respectively).

Of the 122 patients with symptoms of claudication, 18 (14.8%) tested positive for flow obstruction on digital ABI measurement while 54 (11.3%) of the patients without claudication (asymptomatic) tested positive. There was no difference observed in digital ABI value between symptomatic and asymptomatic patients; the mean digital ABI and standard deviation was 0.95±0.13 and 0.96±0.12 for those with claudication (N=122) and without claudication (N=478), respectively.

Table 3 displays prevalence odds ratios for each measure examined. Several risk factors were statistically significantly more common in the PAD positive group than the PAD negative group; these included smoking history (OR 1.63, 95% CI 1.00-2.64), stroke/transient ischemic attack (TIA) (OR 2.70, 95% CI 1.51-4.85), and CAD (OR 1.84, 95% CI 1.12-3.01). From the multivariate analysis, two predictors, hypertension (p=0.018) and stroke/TIA (p=0.0360), emerged as having the most influence on ABI.

**Table 3:** Unadjusted prevalence odds ratios (PAD positive group compared to PAD negative group)

**Measure**	**OR (95% CI)**	**P-value**
Age	1.01 (0.99, 1.03)	0.391
Male	1.54 (0.92, 2.58)	0.099
Claudication	1.36 (0.76, 2.42)	0.295
Discolored/blue legs	1.52 (0.88, 2.63)	0.131
Non-healing wound	1.37 (0.69, 2.74)	0.372
High cholesterol	1.01 (0.61, 1.67)	0.975
Hypertension	0.84 (0.49, 1.44)	0.530
Diabetes	1.32 (0.81, 2.14)	0.266
Smoking history	1.63 (1.00, 2.64)	0.049
Stroke/TIA	2.70 (1.51, 4.85)	<.001
CAD	1.84 (1.12, 3.01)	0.015

## Discussion

In this study, primary care practices evaluated patients suspected to have PAD based on history and physical exam and performed digital ABI measurements. Of these patients, 12% were confirmed to have obstructed flow based on the criteria of the digital ABI test. These results are similar to previous findings using other ABI methods. Reported prevalence of PAD ranges from 4.3% to 16.8%; with age adjusted prevalence of 12% [19-22]. Selvin and Erlinger reported an overall frequency of 4.3%, with a significant increase in prevalence among those 60–69 (4.7%) and over 70 (14.5%) years old [22]. As depicted in Figure 1, our study demonstrated similar trends, with patients 60 years and older yielding the highest frequency of obstructed flow. Characteristics of the patients with obstructed flow identified by digital ABI in this study population were consistent with other PAD studies with respect to mean age, proportion of males, and positive limbs (right, left, or both) [19,20,22].

The prevalence of risk factors in patients with obstructed flow measured by FloChec™ was consistent with previous findings evaluating other techniques. Several studies, including REACH (the largest international PAD registry study conducted to date), report rates of hypercholesterolemia, hypertension, and diabetes (66.7%, 81.0% and 44.6% respectively) similar to or higher than our findings (61.6%, 69.4% and 53.3%, respectively) [19]. Selvin and Erlinger also reported similar rates (60.6%, 73.6% and 26.4%, respectively) [22]. The prevalence of PAD in patients with greater than three risk factors was also concurrent. This study found 65.8% of patients with flow obstruction had three or more risk factors, compared to 60-80% of populations previously studied [19,20,22].

Earlier accurate PAD diagnosis and treatment initiation could potentially greatly improve clinical outcomes; as indicated by Duscha et al., there is a growing body of research around exercise training as therapeutic intervention to promote angiogenesis of the microvasculature in the lower extremity skeletal muscle and improve exercise tolerance [23]. Recent evidence also suggests that a particular calcium/calmodulin-dependent kinase (*CaMK4*) may have an important role in blood pressure regulation through the control of endothelial nitric oxide synthase (eNOS) activity via phosphorylative events. In this study, loss of the *CaMK4* gene resulted in endothelial dysfunction, hypertension, and related complications in mice. In humans, a significant correlation was found between reduced expression of a *CaMK4* polymorphism and higher blood pressure levels among hypertensive patients [9]. Another study on the in vivo effects of a vascular endothelial growth factor (VEGF) mimetic (called QK) offered a potential new method for examining angiogenesis triggered by VEGF receptors. The proteins involved are important for capillary formation and organization and could have large implications for treatment of conditions like chronic ischemia [8]. While research on the etiologies and mechanisms of action of PAD continues, our study supports correlations observed between clinical manifestations and impaired blood flow Figure 1. Interestingly, TIA/stroke (another clinical outcome of endothelial dysfunction) was a statistically significant characteristic in the group found positive for flow obstruction in this study.

Many patients with PAD are asymptomatic and are not referred to vascular specialists when evaluated in the primary care practice. These patients do not begin prophylactic therapies, such as walking, as early as possible. Hirsch et al. found that only 11% of PAD patients had symptoms of claudication [11]. The results of our study suggest slightly higher rates of claudication in the presence of PAD (25%); however, the majority of patients in this group were asymptomatic. Given the increased morbidity and mortality in asymptomatic PAD, specifically without concomitant CAD, and the reported benefits of early treatment, digital ABI could potentially provide early detection and improved disease management [11]. The medical literature indicates that abnormal ABI results are highly concordant with angiographic diagnosis of PAD, supporting the use of ABI or digital ABI in the primary care setting as a more objective assessment of the need for referral to a vascular specialist [24].

Given the severe primary and secondary consequences of delayed diagnosis of PAD, early evaluation and detection is critical. While ABI measurement has long been recognized as an acceptable means of evaluation, traditional Doppler ABI may not be conducive to the primary care setting because this method is time consuming and requires specialized vascular technologists to be performed properly [14]. Development of ABI devices that are easy to use, inexpensive, accurate and less time-consuming can potentially make PAD testing in the primary care practice feasible. The FloChec™ measurement is performed in a few minutes by a medical assistant under the direct supervision of a primary care physician trained on the technique.

Previous studies have found that 46% of patients referred to vascular centers based on clinical suspicion alone were without significant PAD [13]. Our study showed similar results; 41% of patients with a result negative for PAD had presented with at least one sign or symptom. As supported by our research, a significant population presents with clinical symptoms or risk factors that may not be attributable to PAD and who might not require referral to a vascular specialist.

This study had several limitations. The registry was designed to collect and describe digital ABI results and patient history retrospectively, without pre-defined parameters. One major limitation was the lack of direct comparison to Doppler ABI/definitive diagnostic techniques for each observation. Doppler ABI data was not captured as part of the registry database, but is of primary importance for currently ongoing and future studies evaluating this new technology. The passive data collection method used meant that not all data on demographics, signs/symptoms, and risk factors could be collected for all patients. Of the observations provided, 8% had one or more missing data field. All analyses were based on the number of fields completed for each variable of interest.

This was an open data registry; therefore, the clinical sites that provided data did so voluntarily. The variation of clinical care settings allowed for data to be collected in “real world” settings, where a wide variety of patients were tested with the device. As a consequence of this pragmatic approach, the data collection process did not include additional controls or data monitoring. Risks of this method included missing data and possible errors during case report form completion. No inclusion or exclusion criteria were applied; therefore, the data set may be biased in either direction. However, given the large number of patients tested and prevalence rates similar to other studies, the risk of patient selection bias is low. Lastly, this study lacked follow up or definitive diagnostics; therefore, this study does not address sensitivity and specificity of the digital ABI test. Although the results are consistent with previous population-based studies; additional studies are warranted to fully evaluate this technology.

## Conclusions

The results of this large, population-based study support the use of a digital ABI device (FloChec™) using blood volume plethysmography technology for early evaluation and detection of patients at risk for PAD. Compared to Doppler ABI, digital ABI devices enable PAD evaluation in primary care settings and may allow for earlier detection of the disease. Of importance, the Medicare population (over 65 years of age) may benefit most from early evaluation with digital ABI in primary care settings. Further studies are needed to determine the benefit to other high risk populations, such as diabetics, and to evaluate the correlation of digital ABI results using this device with definitive diagnostic techniques.

### Availability of supporting data

The data set supporting the results of this article is included within the article (and its Additional files 1, 2 and 3).

## Abbreviations

ABI: Ankle-brachial index; BFI: Blood flow index; CAD: Coronary artery disease; eNOS: Endothelial nitric oxide synthase; FDA: Food and Drug Administration; LED: Light emitting diode; PAD: Peripheral artery disease; TIA: Transient ischemic attack; VEGF: Vascular endothelial growth factor.

## Competing interests

Gayle Johnson and Tiffini Diage are employed by North American Science Association, which receives payment from Semler Scientific, Inc for database management, data analysis, and medical communication services. Gowtam Ravipati has no competing interests.

## Authors’ contributions

TD developed and coordinated the study and drafted the manuscript. GJ performed the statistical analysis. GR conceived the study and participated in drafting the manuscript. All authors read and approved the final manuscript.

## Supplementary Material

Additional file 1 Full questionnaire administered to patients for evaluation of PAD signs, symptoms, and risk factors. Click here for file

Additional file 2 SAS formats for the dataset, to be used in conjunction with the study dataset. Click here for file

Additional file 3 Dataset containing all study data analyzed and reported on in this article. Click here for file

## References

[B1] Allison MA Ho E Denenberg JO Langer RD Newman AB Fabsitz RR Criqui MH Ethnic-specific prevalence of peripheral arterial disease in the United States Am J Prev Med 2007 32 4 328 333 10.1016/j.amepre.2006.12.010 17383564

[B2] Criqui MH Langer RD Fronek A Feigelson HS Klauber MR McCann TJ Browner D Mortality over a period of 10 years in patients with peripheral arterial disease N Engl J Med 1992 326 6 381 386 10.1056/NEJM199202063260605 1729621

[B3] O’Hare AM Katz R Shlipak MG Cushman M Newman AB Mortality and cardiovascular risk across the ankle-arm index spectrum: results from the Cardiovascular Health Study Circulation 2006 113 3 388 393 10.1161/CIRCULATIONAHA.105.570903 16432070

[B4] Steg PG Bhatt DL Wilson PW D’Agostino R Sr Ohman EM Rother J Liau CS Hirsch AT Mas JL Ikeda Y One-year cardiovascular event rates in outpatients with atherothrombosis JAMA 2007 297 11 1197 1206 10.1001/jama.297.11.1197 17374814

[B5] Ferket BS Spronk S Colkesen EB Hunink MG Systematic review of guidelines on peripheral artery disease screening Am J Med 2011 125 2 198 208 e193 22079018 10.1016/j.amjmed.2011.06.027

[B6] Greenland P Alpert JS Beller GA Benjamin EJ Budoff MJ Fayad ZA Foster E Hlatky MA Hodgson JM Kushner FG ACCF/AHA guideline for assessment of cardiovascular risk in asymptomatic adults: a report of the American College of Cardiology Foundation/American Heart Association Task Force on Practice Guidelines J Am Coll Cardiol 2010 56 25 e50 e103 10.1016/j.jacc.2010.09.001 21144964

[B7] United States Preventative Services Task Force Recommendation Statement: Screening for Peripheral Arterial Disease 2005 Washington, DC: Agency for Healthcare Research and Quality 1 8

[B8] Santulli G Ciccarelli M Palumbo G Campanile A Galasso G Ziaco B Altobelli GG Cimini V Piscione F D’Andrea LD In vivo properties of the proangiogenic peptide QK J Transl Med 2009 7 41 10.1186/1479-5876-7-41 19505323 PMC2702279

[B9] Santulli G Cipolletta E Sorriento D Del-Giudice C Anastasio A Monaco S Maione AS Condorelli G Puca A Trimarco B aMK4 gene deletion induces hypertension J Am Heart Assoc 2012 1 4 e001081 10.1161/JAHA.112.001081 23130158 PMC3487344

[B10] Duscha BD Robbins JL Jones WS Kraus WE Lye RJ Sanders JM Allen JD Regensteiner JG Hiatt WR Annex BH Angiogenesis in skeletal muscle precede improvements in peak oxygen uptake in peripheral artery disease patients Arterioscler Thromb Vasc Biol 2011 31 11 2742 2748 10.1161/ATVBAHA.111.230441 21868709 PMC3578302

[B11] Hirsch AT Criqui MH Treat-Jacobson D Regensteiner JG Creager MA Olin JW Krook SH Hunninghake DB Comerota AJ Walsh ME Peripheral arterial disease detection, awareness, and treatment in primary care JAMA 2001 286 11 1317 1324 10.1001/jama.286.11.1317 11560536

[B12] Nelson MR Quinn S Winzenberg TM Howes F Shiel L Reid CM Ankle-Brachial Index determination and peripheral arterial disease diagnosis by an oscillometric blood pressure device in primary care: validation and diagnostic accuracy study BMJ Open 2 5 pii: e001689. doi: 10.1136/bmjopen-2012-001689. Print 2012. 10.1136/bmjopen-2012-001689 PMC3488728 23100446

[B13] Sillesen H Falk E Peripheral artery disease (PAD) screening in the asymptomatic population: why, how, and who? Curr Atheroscler Rep 2011 13 5 390 395 10.1007/s11883-011-0196-x 21811798

[B14] Premanath M Raghunath M Ankle-Brachial index by oscillometry: a very useful method to assess peripheral arterial disease in diabetes Int J Diabetes Dev Ctries 30 2 97 101 20535314 10.4103/0973-3930.62600 PMC2878698

[B15] Jonsson B Laurent C Eneling M Skau T Lindberg LG Automatic ankle pressure measurements using PPG in ankle-brachial pressure index determination Eur J Vasc Endovasc Surg 2005 30 4 395 401 10.1016/j.ejvs.2005.05.012 15964772

[B16] Khandanpour N Armon MP Jennings B Clark A Meyer FJ Photoplethysmography, an easy and accurate method for measuring ankle brachial pressure index: can photoplethysmography replace Doppler? Vasc Endovascular Surg 2009 43 6 578 582 10.1177/1538574409334829 19640917

[B17] Sadiq S Chithriki M Arterial pressure measurements using infrared photosensors: comparison with CW Doppler Clin Physiol 2001 21 1 129 132 10.1046/j.1365-2281.2001.00299.x 11168307

[B18] Whiteley MS Fox AD Horrocks M Photoplethysmography can replace hand-held Doppler in the measurement of ankle/brachial indices Ann R Coll Surg Engl 1998 80 2 96 98 9623371 PMC2502998

[B19] Bhatt DL Steg PG Ohman EM Hirsch AT Ikeda Y Mas JL Goto S Liau CS Richard AJ Rother J International prevalence, recognition, and treatment of cardiovascular risk factors in outpatients with atherothrombosis JAMA 2006 295 2 180 189 10.1001/jama.295.2.180 16403930

[B20] Diehm C Schuster A Allenberg JR Darius H Haberl R Lange S Pittrow D von Stritzky B Tepohl G Trampisch HJ High prevalence of peripheral arterial disease and co-morbidity in 6880 primary care patients: cross-sectional study Atherosclerosis 2004 172 1 95 105 10.1016/S0021-9150(03)00204-1 14709362

[B21] Hiatt WR Medical treatment of peripheral arterial disease and claudication N Engl J Med 2001 344 21 1608 1621 10.1056/NEJM200105243442108 11372014

[B22] Selvin E Erlinger TP Prevalence of and risk factors for peripheral arterial disease in the United States: results from the National Health and Nutrition Examination Survey, 1999–2000 Circulation 2004 110 6 738 743 10.1161/01.CIR.0000137913.26087.F0 15262830

[B23] Duscha BD Robbins JL Kontos CD Kraus WE Annex BH Santulli G Angiogenesis in peripheral artery disease: an emerging therapy targeting skeletal muscle Angiogenesis: Insights From a Systematic Overview 2013 New York: Nova Publishers 99 134

[B24] Dachun X Jue L Liling Z Yawei X Dayi H Pagoto SL Yunsheng M Sensitivity and specificity of the ankle--brachial index to diagnose peripheral artery disease: a structured review Vasc Med 15 5 361 369 10.1177/1358863X10378376 20926495

