# Supplementary material for: Digital ankle-brachial index technology used in primary care settings to detect flow obstruction: a population based registry study
Source: BMC Res Notes. 2013 Oct 8;6:404. doi: 10.1186/1756-0500-6-404 (PMC3876723; doi:10.1186/1756-0500-6-404)
Supplement: Additional file 1 — Full questionnaire administered to patients for evaluation of PAD signs, symptoms, and risk factors. [file 1756-0500-6-404-S1.pdf]

Do I Need a Test for PAD?

Peripheral Arterial Disease (PAD) is a serious circulatory problem in which the blood vessels that carry blood to your arms, legs, brain, or kidneys, become narrowed or clogged. It affects over 18 million Americans, most over the age of 50. People with PAD are at significantly increased risk for stroke and heart attack. Answers to these questions will determine if you are at risk for PAD and if a vascular exam will help us better assess your vascular health status.

Print Initials:______ Age:______ Today's Date:______

Circle "Yes" or "No":

1. Do you experience any pain at rest in your lower leg(s) or feet?	Yes	No
2. Do you have foot, calf, buttock, hip or thigh discomfort (aching, fatigue, tingling, cramping or pain) when you walk which is relieved by rest?	Yes	No
3. If yes to #2, does the pain go away within 10-minutes after stopping?	Yes	No
4. Are your toes or feet pale, discolored, or bluish?	Yes	No
5. Do you have an infection, skin wound or ulcer on your feet or toes that are slow to heal (8-12 weeks)?	Yes	No
6. Do you have a high cholesterol level or other blood lipid problem or take medication to lower the cholesterol level?	Yes	No
7. Do you have high blood pressure or take medication for high blood pressure?	Yes	No
8. Do you have diabetes?	Yes	No
9. Have you ever smoked?	Yes	No
10. Have you previously had a stroke?	Yes	No
11. Do you have heart disease?	Yes	No

Left dABI: [ ]   Right dABI: [ ]

Nurse's Signature: ________________________ Time: _________ Date: __________

Physician Signature: ________________________________ Date: ________________
